# The Human Brain: Its Structure, Physiology, and Diseases. With a Description of the Typical Forms of Brain in the Animal Kingdom

**Published:** 1847-10

**Authors:** 


### ART. XIX.

*The Human Brain: its Structure, Physiology, and Diseases. With a Description of the Typical Forms of Brain in the Animal Kingdom.* By SAMUEL SOLLY, F.R.S., Senior Assistant-Surgeon to St. Thomas's Hospital, and Lecturer on Clinical Surgery, &c. &c. With numerous Wood-Engravings. Second Edition.—*London,* 1847. 8vo, pp. 688.

THIS very beautiful volume might almost be considered as a new work, rather than as a new edition of the treatise published by Mr. Solly under the same title eleven years since, and noticed by us with much commendation in our Eighth Number, Its size has been greatly augmented; the whole of its matter has been remodelled; and the lithographic plates of the original have been superseded by no fewer than 118 wood-engravings, many of them of the most elaborate character, and all executed in the first style of art. We regret that our limits compel us to present our readers with a much more cursory notice of its contents than their important character might fairly claim; and in justice to Mr. Solly we shall chiefly direct attention to those features of his work which give to it its most distinctive character.

The first chapter gives a sufficiently ample description, derived from the most recent and accurate sources, of the elementary structures of which the nervous system is composed. Mr. Solly may very reasonably lay claim to the merit of having been among the first to entertain that view of the functional relations between the white and gray, or the tubular and vesicular forms of neurine, which is now almost universally received amongst physiologists; having expressed himself quite clearly and decidedly on this point in his first edition. Evidence in favour of this doctrine has since been furnished from so many different sources, and it seems altogether so firmly based, that we *now* are tempted to overlook its very recent origin, and to wonder that it should have been ever doubted.

In the second chapter, we find such a general *resumé* of the comparative anatomy of the nervous system, as is calculated to prepare the student for the due comprehension of its structure in man. Commencing with the starfish, he shows that we find in it the elements of the most highly developed nervous system; namely, ganglionic centres, commissures, and nerves. The brain of man is nothing else than a collection of distinct ganglionic centres, packed together in one mass; its complexity of structure depending upon the number of these which the different functions require, and upon the intimacy of relation which is established between them by the various commissures. Mr. Solly proposed, in his former edition, the term *hemispherical ganglia* to distinguish the cortical substance of the cerebral hemispheres from the other masses of gray matter which are included under the collective designation Brain, or even within the more limited term Cerebrum; and although it is rather too lengthy for ready use, we do not know what other could be advantageously substituted for it. His early perception of the fact, that the various insulated patches of gray matter at the base of the cerebrum and in the medulla oblongata must be regarded as so many distinct and independent ganglia, each of which has its own proper function, was, in our apprehension, the great merit of his original descriptions; and it has been his guide in various subsequent anatomical inquiries, the results of which we shall presently notice. After a brief general exposition of the nature and offices of the principal divisions of the nervous system, Mr. Solly traces these through the higher invertebrata; quoting the anatomical descriptions of Garner and Owen for the mollusca, and of Newport for the articulata; and adopting the physiological explanations given by Dr. Carpenter in his Prize Thesis and subsequent writings. It is to the anatomy of the nervous system in the vertebrated classes that Mr. Solly has more particularly devoted his own attention; and here we find the descriptions drawn almost entirely from his own observations, although he has occasionally profited by the writings of Serres, Leuret, Owen, and others. In attributing to Professor Owen and to Mr. Newport, in a former article, the first indication of the true homology of the cephalic ganglia of the mollusca and articulata respectively, we had overlooked the fact that Mr. Solly had previously stated, in general but distinct terms, his opinion that the hemispheric ganglia are organs confined to the vertebrata, and that the cephalic ganglia of the invertebrata are collective representatives of the ganglia in immediate connexion with the organs of sense, which make up the principal part of the encephalon of the lower fishes. This is another instance of Mr. Solly's clear-sightedness; and we gladly accord him the credit of having been, as we believe, the first to form a stable and permanent bridge over the chasm that was previously supposed to divide the encephalic centres of the vertebrated from those of the invertebrated animals. If, as we believe, our future advances in the physiology of the brain depend more upon a philosophical use of the facts supplied by comparative anatomy, than upon any other single source of information, we shall owe much to Mr. Solly for having thus indicated the right path of inquiry.

At the time when Mr. Solly's first edition was published, anatomists and physiologists were far from being agreed respecting the homology of the fish's brain; and many mistakes had been made, in consequence of insufficient scrutiny into the connexions of the elements of which it is composed. Where *four* ganglia were present in approximation with each other in the antero-posterior direction, as in the encephalon of the eel or pike, the most anterior, from its evident connexion with the olfactory nerves, had been recognised as the olfactory ganglion, whilst the hindmost had been universally and correctly designated as the cerebellum. But of the two intermediate ganglia, the anterior were regarded by some, and the posterior by others, as the homologues of the cerebral hemispheres of higher animals. And the discrepancy was still further increased by the misinterpretation of the structure of the encephalon of the cod, whiting, &c., in which the olfactory ganglia are removed to a great distance from the posterior masses, and are connected with them by long peduncles. The existence of these olfactory ganglia was overlooked, and the commissural peduncles were treated as the olfactory nerves; so that the total number of ganglionic masses in such cases was only reckoned at three, and what are really the second pair of ganglia in the fish's brain (reckoning from before backwards) were frequently taken for the first, or were supposed to combine the functions of the first and second. To Mr. Solly is due the chief merit of having corrected this important error, into which an anatomist no less distinguished than Serres had fallen, and from which not even Leuret (who does not seem to have been acquainted with Mr. Solly's work) has escaped. It was shown by Mr. Solly that in no instance are there fewer than four distinct ganglionic masses in the fish's encephalon; and that there always exists a pair of olfactive ganglia anterior to those which constitute the rudiment of the cerebral hemispheres, although the distance between them is frequently very considerable. Thus in a carp, whose entire length was but seven inches, the length of the commissural peduncle was somewhat above an inch. The encephalon of the carp is one of the most interesting of all the brains of this class of animals; from the circumstance that certain ganglionic centres, which have elsewhere coalesced or are buried in the medulla oblongata, are here distinct. This is the case with the auditory ganglia, whose homologues in man, as we shall presently see, are the gray nuclei of the posterior pyramidal bodies; and also with the pneumogastric ganglia, which are represented in the human subject by the gray matter of the restiform bodies.

Having so recently expressed our convictions as to the impossibility of obtaining a correct idea of the component parts of the human encephalon, without a careful study of the ascending series of brains in which successive stages of complication are exhibited, we need not now follow Mr. Solly through his detailed description of the comparative anatomy of this organ in reptiles, birds, and mammals; but shall only say that it exhibits throughout the clearness and completeness which mark a thorough practical acquaintance with his subject, and the conciseness and simplicity which are indicative of ample experience in anatomical tuition; whilst the details are never obtrusively presented, as they are in most works treating *ex professo* on comparative anatomy, but are all kept in subordination to the one great object, the illustration of the structure of the human brain. But we shall direct the attention of our readers to his account of the structure of the medulla oblongata, which he has made the subject of careful and repeated investigation, both in man and the lower animals. It is, in fact, first brought under consideration when the brain of the sheep is being described. No mistake is more common, than to confuse the *strands* or bundles of fibres which go under the names of corpora pyramidalia (anteriora and posteriora), olivaria, and restiformia, with the *nuclei* of gray matter which some of these contain, but with which functionally they have nothing to do. The strands are cords of communication between the encephalon above and the spinal cord below; whilst the nuclei are the ganglionic centres of the nerves issuing from the medulla oblongata itself. The anterior pyramids are *merely* fibrous tracts; but the olivary, restiform, and posterior pyramidal bodies possess gray nuclei imbedded in these. The size of these projections on the surface of the medulla oblongata bears no relation whatever to that of the nuclei; and the absence of the latter must not be inferred from the want of the former. Thus the olivary bodies have been affirmed to have no existence in many mammalia, their highest development being attained in man. Mr. Solly has found the ganglionic nuclei, however, in the sheep, horse, calf, and cat; and he entertains no doubt that they are universally present. Their position in the horse is curiously changed, as they approximate much more closely to the median line, so as almost to occupy the place of the pyramids in man; so that, as Mr. Solly justly remarks, they must be regarded as ganglia simply imbedded in the motor tract, and as not forming any line of physiological demarcation. In his former edition he had expressed the idea that they might be regarded as the ganglionic centres of the pneumogastric nerve; but his later researches have shown him that this was an erroneous determination, and that the real pneumogastric ganglia are those imbedded in the restiform bodies, whilst the olivary ganglia are the centres of the motor nerves of the tongue, commonly termed the ninth pair. The great bulk of the vesicular nervous substance which they contain in man would thus seem to correspond with the multiplied movements of his tongue as an organ of speech.

The gray nucleus of the restiform body, or the proper restiform ganglion, had been recognised by Stilling as the true centre of the pneumogastric and glossopharyngeal nerves; but he had not sufficiently distinguished it from the auditory ganglion or nucleus of the posterior pyramidal body, with which it is in close contiguity. The clear determination of this point, which may be more distinctly made out in some of the lower mammalia than in man, is due to Mr. Solly; who has thus had a large share in the discovery of another very important fact in physiological anatomy. It may be interesting to remark that this close connexion of the auditory ganglion with the pneumogastric harmonizes well with the direct influence which the sensations derived through the former have upon the movements of vocalization, in which the latter has so considerable a share. In the locus niger, a collection of gray matter in the substance of the crus cerebri, to which no one, we believe, had previously assigned a definite function, Mr. Solly finds the ganglionic centre of the third or oculo-motor nerve, one root of which passes into it.

Our readers are probably aware that to Mr. Solly is due the discovery of the connexion between the anterior columns and the cerebellum by three sets of fibres, of which two are superficial, and are derived from the pyramidal columns, whilst another is deep, and is derived from the olivary columns. The former had been previously noticed by Santorini and Rolando, under the name of the "arciform" fibres; but these anatomists do not seem to have been aware that these fibres passed directly to the cerebellum by the restiform column with which they become incorporated, but described them as passing down from the pons varolii. The merit of Mr. Solly's researches on this head consists in his having traced out the superior connexions of these superficial fibres, which Rolando had left obscure; and also in having discovered an entirely new and deeper series connecting the cerebellum and the olivary columns. Another very interesting point in the anatomy of the medulla oblongata seems to have been settled by his researches; namely, the decussation of the sensory columns. The existence of this might be predicated upon physiological grounds, from phenomena of the same class with those which led the decussation of the motor columns to be anticipated; but though Sir C. Bell thought that he had made it out, it has not been generally admitted as an anatomical fact. According to Mr. Solly, the decussation does not take place at a point so low as that described by Sir C. Bell; and the last-named anatomist was probably misled by the appearance presented by the posterior part of the motor column, where the anterior pyramids are interlacing. The decussation seen by Mr Solly takes place whilst the sensory tract is passing through the pons varolii, and is consequently above the origin of the auditory nerve. It appears from Foville's delineations, that he had previously recognised it; but he does not describe it. The same decussation has been recently figured by Dr. Radclyffe Hall in the plates illustrating the last of his papers on the 'Sympathetic Nerve' (Edinb. Med. and Surg. Journal, July, 1847, Plate VII); but we do not find any detailed description of it in his text.

On one very important anatomical question,—namely, the connexion of the roots of the spinal nerves with the cord,—Mr. Solly gives us valuable testimony from his own investigations. Our readers will be aware that a few years ago the prevalent idea was, that all the roots of the spinal nerves are continuous with the white or fibrous strands of the cord; so that when Mr. Grainger first ventured to assert that a portion of each root terminates in the gray matter of the cord, his statement was received with considerable hesitation. Of late, however, the current has set in the opposite direction; for Stilling and Messrs. Todd and Bowman have been disposed to deny that the continuity of any of the fibres entering into the roots of the nerves with the fibrous columns of the cord can be distinctly traced; and they have thus considered the gray matter of the spinal cord as the ganglionic centre of *all* the roots of the spinal nerves; its white columns being commissural in their character, conveying influence from one ganglionic centre to another, instead of establishing a direct connexion between the encephalon and certain portions of each spinal nerve. We have never been able to regard this view as physiologically probable; and it has always appeared to us inconsistent with the analogy of the articulata, in which the direct connexion of the nerves of the cord with the cephalic ganglia, through the medium of the fibrous tract, is sufficiently clear. Dr. Budge of Bonn had contested the accuracy of Stilling's assertions on this point; and we are happy to find that Mr. Solly's observations fully coincide with his,—fully bearing out the truth of Mr. Grainger's original statement.

We have thought it due to Mr. Solly, and likely to interest our readers, that we should thus briefly advert to the chief points on which his anatomical labours have contributed to the positive advance of neurology. The value of this portion of his work, however, does not depend upon the number of new facts it contains, but upon the aspect in which it has placed the old. We have not space to follow him through his account of the anatomy of the human brain, towards which all the previous details of comparative anatomy are made skilfully to converge; but we shall quote the concluding paragraphs, which will show the general plan on which his descriptions are arranged.

"The above descriptions demonstrate that the encephalon or brain in the human subject is not a large solid mass of matter, in the interior of which are cavities scooped, as it were, out of its substance to be appropriately denominated ventricles; but that it really consists of ganglia or collections of cineritious neurine, placed on each side of the mesial line:—some of them being the appropriate ganglia of the nerves of sensation, as, for instance, the olfactory ganglia, the optic ganglia or tubercula quadrigemina, the auditory ganglia or posterior pyramidal bodies, the pneumogastric ganglia or restiform ganglia, the olivary bodies or lingual ganglia; the others being the motory and sensory ganglia, as the corpora striata and thalami nervorum opticorum. The hemispherical ganglia, again, that they might present the greatest possible extent of surface, are folded up into innumerable plaits, and thus cover or surround every other ganglion within the cranium, so that, on first removing the skull-cap, nothing can be seen but the convoluted surface of these extensive ganglia.

"And here let me insist upon this important principle in the study of the brain, which is also one of the first ideas that the student should acquire regarding its composition, namely, that it consists of corresponding or symmetrical parts on each side of the mesial plane; and that, instead of regarding the fissures of separation between its different portions as forming ventricles or cavities, he must direct his attention to the ganglia which bound the fissure, and to the structures called commissures, which, connecting them together, cross the fissure, and necessarily alter its character in different points, masking it, it is true, but not at any place changing the fissure into a true bag or circumscribed cavity. The third, the iter a tertio ad quartum ventriculum, the fourth, and fifth ventricles, as we have already seen, are in truth no more than the successive dilatations from below upwards of the posterior fissure of the cord; difficult enough to be understood when these are viewed in different situations and unconnected with each other, as in the ordinary mode of dissecting the brain, but which seem necessary and obvious where its parts are traced in connexion with one another.

"In conclusion, let me express the hope that these views or analyses, if I may be allowed so to call them, of the component parts of the encephalon, will really simplify the whole of its anatomy, and materially assist the student in acquiring a knowledge of its true character; I wish that custom did not require the student to burthen his memory with fanciful and unmeaning names, and that instead of learning a long catalogue of the contents of the lateral ventricles, as they are erroneously designated, and puzzling himself with the absurd titles of hippocampus major and minor, pes hippocampi, tænia hippocampi, cornu ammonis, &c., he should be required simply to observe how the spinal columns appear to terminate superiorly in two large tubercles, the *corpora striata* and *thalami*, from the sides and under parts of which the hemispheres spring out, being afterwards reflected so as completely to envelop this bulbous extremity of the spinal cord. In the same way, the third ventricle should be described as a fissure separating the two halves of the brain, his particular attention being directed to the commissures which pass across it to connect the different cerebral ganglia with one another. The description of the relative position of these ganglia, the commissures connecting them, and their relation to the ganglia and columns of the spinal cord, comprehend all the information which is either interesting or useful to the student." (p. 283.)

We regret extremely that our vanished space compels us to omit all notice of the pathological part of this volume. We can, however, assure our readers that they will find in it much valuable practical matter. The work altogether reflects the highest credit on the author, and ought to find a place in the library of every practitioner.

